# Impact of postoperative fluorodeoxyglucose positron emission tomography/computed tomography on adjuvant head and neck cancer treatment

**DOI:** 10.1093/jncics/pkaf077

**Published:** 2025-07-23

**Authors:** P Travis Courtney, Jesus E Juarez Casillas, Eulanca Y Liu, Myung-Shin Sim, Lydia W Chau, Rafael E Lopez-Chicas, Maie A St John, Elliot Abemayor, Keith E Blackwell, Dinesh K Chhetri, Quinton S Gopen, Paul A Kedeshian, Rhorie P Kerr, Jivianne K Lee, Vishad Nabili, Joel A Sercarz, Jeffrey D Suh, Marilene B Wang, Deborah J Wong, Wanxing Chai-Ho, Mahbod G Jafarvand, Shadfar Bahri, Erika Jank, Vishruth K Reddy, Michael L Steinberg, Robert K Chin, Ricky R Savjani

**Affiliations:** Department of Radiation Oncology, University of California, Los Angeles, Los Angeles, CA, United States; Head and Neck Cancer Program, University of California, Los Angeles, Los Angeles, CA, United States; Department of Radiation Oncology, University of California, Los Angeles, Los Angeles, CA, United States; Head and Neck Cancer Program, University of California, Los Angeles, Los Angeles, CA, United States; Department of Radiation Oncology, University of California, Los Angeles, Los Angeles, CA, United States; Head and Neck Cancer Program, University of California, Los Angeles, Los Angeles, CA, United States; Department of Medicine Statistics Core, Division of General Internal Medicine and Health Services Research, University of California, Los Angeles, Los Angeles, CA, United States; Department of Radiation Oncology, University of California, Los Angeles, Los Angeles, CA, United States; Head and Neck Cancer Program, University of California, Los Angeles, Los Angeles, CA, United States; Department of Radiation Oncology, University of California, Los Angeles, Los Angeles, CA, United States; Head and Neck Cancer Program, University of California, Los Angeles, Los Angeles, CA, United States; Head and Neck Cancer Program, University of California, Los Angeles, Los Angeles, CA, United States; Department of Otolaryngology-Head and Neck Surgery, University of California, Los Angeles, Los Angeles, CA, United States; Head and Neck Cancer Program, University of California, Los Angeles, Los Angeles, CA, United States; Department of Otolaryngology-Head and Neck Surgery, University of California, Los Angeles, Los Angeles, CA, United States; Head and Neck Cancer Program, University of California, Los Angeles, Los Angeles, CA, United States; Department of Otolaryngology-Head and Neck Surgery, University of California, Los Angeles, Los Angeles, CA, United States; Head and Neck Cancer Program, University of California, Los Angeles, Los Angeles, CA, United States; Department of Otolaryngology-Head and Neck Surgery, University of California, Los Angeles, Los Angeles, CA, United States; Head and Neck Cancer Program, University of California, Los Angeles, Los Angeles, CA, United States; Department of Otolaryngology-Head and Neck Surgery, University of California, Los Angeles, Los Angeles, CA, United States; Head and Neck Cancer Program, University of California, Los Angeles, Los Angeles, CA, United States; Department of Otolaryngology-Head and Neck Surgery, University of California, Los Angeles, Los Angeles, CA, United States; Head and Neck Cancer Program, University of California, Los Angeles, Los Angeles, CA, United States; Department of Otolaryngology-Head and Neck Surgery, University of California, Los Angeles, Los Angeles, CA, United States; Head and Neck Cancer Program, University of California, Los Angeles, Los Angeles, CA, United States; Department of Otolaryngology-Head and Neck Surgery, University of California, Los Angeles, Los Angeles, CA, United States; Head and Neck Cancer Program, University of California, Los Angeles, Los Angeles, CA, United States; Department of Otolaryngology-Head and Neck Surgery, University of California, Los Angeles, Los Angeles, CA, United States; Head and Neck Cancer Program, University of California, Los Angeles, Los Angeles, CA, United States; Department of Otolaryngology-Head and Neck Surgery, University of California, Los Angeles, Los Angeles, CA, United States; Head and Neck Cancer Program, University of California, Los Angeles, Los Angeles, CA, United States; Department of Otolaryngology-Head and Neck Surgery, University of California, Los Angeles, Los Angeles, CA, United States; Head and Neck Cancer Program, University of California, Los Angeles, Los Angeles, CA, United States; Department of Otolaryngology-Head and Neck Surgery, University of California, Los Angeles, Los Angeles, CA, United States; Head and Neck Cancer Program, University of California, Los Angeles, Los Angeles, CA, United States; Department of Hematology Oncology, University of California, Los Angeles, Los Angeles, CA, United States; Head and Neck Cancer Program, University of California, Los Angeles, Los Angeles, CA, United States; Department of Hematology Oncology, University of California, Los Angeles, Los Angeles, CA, United States; Department of Nuclear Medicine, University of California, Los Angeles, Los Angeles, CA, United States; Department of Nuclear Medicine, University of California, Los Angeles, Los Angeles, CA, United States; Department of Radiation Oncology, University of California, Los Angeles, Los Angeles, CA, United States; Physics and Biology in Medicine, Graduate Programs in Bioscience, University of California, Los Angeles, Los Angeles, CA, United States; Department of Radiation Oncology, University of California, Los Angeles, Los Angeles, CA, United States; Head and Neck Cancer Program, University of California, Los Angeles, Los Angeles, CA, United States; Department of Radiation Oncology, University of California, Los Angeles, Los Angeles, CA, United States; Head and Neck Cancer Program, University of California, Los Angeles, Los Angeles, CA, United States; Department of Radiation Oncology, University of California, Los Angeles, Los Angeles, CA, United States; Head and Neck Cancer Program, University of California, Los Angeles, Los Angeles, CA, United States; Department of Radiation Oncology, University of California, Los Angeles, Los Angeles, CA, United States; Head and Neck Cancer Program, University of California, Los Angeles, Los Angeles, CA, United States

## Abstract

**Background:**

Residual or recurrent cancer after surgery but prior to adjuvant therapy occurs in a proportion of patients with head and neck cancer and may warrant treatment changes. 18-Fluorodeoxyglucose positron emission tomography/computed tomography (FDG-PET/CT) may help to identify residual or recurrent disease but is not routinely obtained. We evaluated the relevance of postoperative FDG-PET/CT in this clinical context.

**Methods:**

This single-institution, retrospective study identified patients with head and neck cancer who underwent definitive surgery between January 1, 2013, and April 1, 2023, and received a postoperative FDG-PET/CT prior to adjuvant treatment. We measured the rates of management changes resulting from postoperative FDG-PET/CT findings and the association between having a postoperative FDG-PET/CT which resulted in a management change and oncologic outcomes with selected multivariable competing-risks and proportional hazards regressions.

**Results:**

Of 150 patients, 66 (44.0%) had a management change because of the postoperative FDG-PET/CT findings; 62 (93.8%) had radiotherapy plan changes, 20 (30.3%) underwent additional diagnostic testing, 11 (16.7%) had systemic therapy added or changed, 3 (4.6%) underwent reresection, and 15 (10.0%) switched to palliative-intent treatment. Having a postoperative FDG-PET/CT that resulted in a management change was not significantly associated with cancer recurrence or overall survival (both *P *> .05).

**Conclusions:**

In patients with resected head and neck cancer, postoperative, pre-adjuvant therapy FDG-PET/CT can alter clinical management and may enable additional personalization of treatment. When practical to obtain without delaying treatment, postoperative FDG-PET/CT may have clinical utility though requires careful interpretation due to the risks of false positives.

## Introduction

Surgical resection followed by adjuvant radiotherapy with or without systemic therapy is a standard of care treatment strategy for many patients with head and neck cancer.^1^ Even within the brief recommended interval between surgery and adjuvant therapy,^1^^,^^2^ a proportion of patients can experience rapidly progressive residual or recurrent disease.^3-8^ Identification of such occurrences can prompt changes in adjuvant management that enable further personalization of treatment that may ultimately improve clinical care. Obtaining an 18-fluorodeoxyglucose positron emission tomography/computed tomography (FDG-PET/CT) postoperatively but prior to adjuvant therapy can identify residual or early recurrent cancer^3-6^ and may be prognostic and improve outcomes in certain patients.^3-5^

Despite the possible benefits of detecting residual or early recurrent disease using postoperative FDG-PET/CT, routine clinical practice does not currently include obtaining a postoperative, pre-adjuvant therapy FDG-PET/CT. Besides the logistical challenges of obtaining an FDG-PET/CT in the recommended time between surgery and adjuvant therapy, there are concerns regarding its interpretability in the early postoperative period.^9^^,^^10^ There are limited data on the utility of FDG-PET/CT in this context. Herein, we evaluate the clinical relevance of postoperative FDG-PET/CT prior to adjuvant therapy in patients with resected head and neck cancer.

## Methods

### Study population

We used an institutional database comprising claims-based and structured electronic medical record interface data to identify patients for this study. Templated medical system encounter data was used to identify patients with head and neck cancer who underwent definitive surgery between January 1, 2013, and April 1, 2023, at our institution followed by a postoperative FDG-PET/CT and adjuvant radiotherapy. Postoperative FDG-PET/CT and adjuvant therapy could occur at any point after surgery so long as FDG-PET/CT occurred prior to initiation of adjuvant radiotherapy. Identified patients were then manually verified via chart review to fit the preceding criteria. Waivers of consent and authorization were granted by the institutional review board (institutional review board protocol #18-001562).

### Outcomes

The primary outcome is the rates of clinical management changes resulting from postoperative FDG-PET/CT findings. We further characterized management changes as follows: (1) obtaining additional diagnostic testing, such as a biopsy or additional imaging; (2) changes to the anticipated adjuvant radiotherapy plan, such as increasing the dose (“boosting”) or targeting a new area; (3) the addition of or change in anticipated systemic therapy; (4) undergoing repeat surgery; and (5) switching to palliative-intent treatment. Patients could experience one or multiple types of management changes. We determined if a change in management occurred and the type through manual chart review and evaluation of radiation treatment plans. Additionally, for changes in radiotherapy plans, we compared what the radiotherapy plan would have been, based on surgical pathology and other clinical factors prior to postoperative FDG-PET/CT, with the ultimate delivered radiotherapy. The presence and type of management changes were reviewed by an attending head and neck radiation oncologist (R.K.C. and R.R.S.).

We considered a patient as having a “positive” postoperative FDG-PET/CT if their postoperative FDG-PET/CT findings resulted in a management change. All patients who were included had indications for adjuvant radiotherapy and were not being considered for treatment deintensification based on postoperative FDG-PET/CT results. FDG-PET/CTs were interpreted in conjunction by physicians within our institution’s departments of radiology and nuclear medicine, and subsequent managements changes were determined at the treating physician’s or physicians’ discretion, often after multidisciplinary discussion.

Secondary outcomes included the association between various patient characteristics and the likelihood of having a positive postoperative FDG-PET/CT and the association between oncologic outcomes and having a positive postoperative FDG-PET/CT. Oncologic outcomes were locoregional or distant recurrence without death and overall survival.

### Statistical analysis

We extracted electronic medical record data supplemented with manual chart review to collect relevant patient information. Two-sample *t*-tests, Pearson χ^2^, and Wilcoxon rank-sum tests were used to assess differences in baseline covariates. We measured the association of patient covariates with the likelihood of their postoperative FDG-PET/CT findings resulting in a management change with univariable followed by selected multivariable logistic regression. The covariate selection process is described in the Supplementary Methods. The Hosmer–Lemeshow test demonstrated good fit for the selected multivariable logistic regression model (*P *= .20), and this model was validated by bootstrap resampling with 500 samples. Follow-up was measured from surgery to last follow-up or death. Follow-up cutoff was April 30, 2024. Recurrence was measured from end of radiotherapy to date of radiographic or pathologic evidence, whichever occurred first.

The Fine–Gray competing risks model was used to handle the presence of competing risks when assessing the association between having a positive postoperative FDG-PET/CT and cancer recurrence.^11^ In this context, competing risks are events that preclude the occurrence of the primary event of interest; in this case, death from any cause before recurrence can be observed. The association between overall survival and having a positive postoperative FDG-PET/CT was measured using Cox proportional hazards regression. Selection of covariates for the multivariable Fine–Gray and Cox regression models is described in the Supplementary Methods. We measured the rates of recurrence and all-cause mortality with cumulative incidence functions.^12^

In a sensitivity analysis, we performed the logistic, competing-risks, and Cox regression analyses in only patients with squamous cell carcinoma (SCC) histology. The proportional hazards assumption was tested through visual inspection of Kaplan-Meier curves and no violations were seen. We used SAS V9.4 (SAS Institute, Cary, NC) for all statistical analyses, assuming a 2-sided alpha of 0.05. Figures were designed with RStudio 2024.04.2 (RStudio, Boston, MA, United States).

## Results

### Study population

We identified 150 patients with head and neck cancer who received a postoperative FDG-PET/CT prior to adjuvant therapy with a median follow-up of 21.2 months (interquartile range [IQR] = 12.5-46.1). Cohort description is provided in Table 1. The most common primary site was oral cavity (*n* = 46, 30.7%), and the most common histology was SCC (*n* = 93, 62.0%); 52% of patients had pT0-2 disease, and 71.3% had pNx-1 disease. Moreover, 41 (27.3%) patients presented with recurrent disease; 87 (58.0%) patients received preoperative staging imaging, defined as an FDG-PET/CT or CT chest within 6 months prior to surgery, the majority of which were FDG-PET/CT (*n* = 79, 90.88%). The median time from surgery to postoperative FDG-PET/CT was 35.5 days (IQR = 26.0-51.0) and from surgery to start of adjuvant radiotherapy was 54.0 days (IQR = 43.0-69.0).

**Table 1.** Cohort demographics.

Variable	All patients (*n* = 150)	No management change (*n* = 84, 56.0%)	Management change (*n* = 66, 44.0%)	*P* ^a^
Age at surgery, mean (SD), years	64.5 (15.4)	64.3 (15.1)	64.8 (15.8)	.84
Male sex, no. (%)	102 (68.0)	55 (65.5)	47 (71.2)	.45
Self-reported race, no. (%)				.68
Asian	24 (16.0)	14 (16.7)	10 (15.2)	
Not reported	17 (11.3)	9 (10.7)	8 (12.1)	
Other^b^	41 (27.3)	20 (23.8)	21 (31.8)	
White	68 (45.3)	41 (48.8)	27 (40.9)	
Self-reported ethnicity, no. (%)				.13
Hispanic	23 (15.3)	11 (13.1)	12 (18.2)	
Non-Hispanic	117 (78.0)	70 (83.3)	47 (71.2)	
Not reported	10 (6.7)	3 (3.6)	7 (10.6)	
Ever tobacco use, no. (%)	62 (41.3)	32 (38.1)	30 (45.4)	.36
Year of surgery, no. (%)				.049
2013	5 (3.3)	4 (4.8)	1 (1.5)	
2014	8 (5.3)	7 (8.3)	1 (1.5)	
2015	3 (2.0)	3 (3.6)	0 (0.0)	
2016	7 (4.7)	3 (3.6)	4 (6.1)	
2017	9 (6.0)	5 (5.9)	4 (6.1)	
2018	9 (6.0)	5 (5.9)	4 (6.1)	
2019	17 (11.3)	12 (14.3)	5 (7.6)	
2020	16 (10.7)	6 (7.1)	10 (15.1)	
2021	29 (19.3)	17 (20.4)	12 (18.2)	
2022	38 (25.3)	21 (25.0)	17 (25.8)	
2023	9 (6.0)	1 (1.2)	8 (12.1)	
Preoperative staging imaging, no. (%)	87 (58.0)	40 (47.6)	47 (71.2)	.004
Primary site, no. (%)				.10
Oral cavity^c^	46 (30.7)	19 (22.6)	27 (40.9)	
Oropharynx	21 (14.0)	12 (14.3)	9 (13.6)	
Salivary gland	18 (12.0)	14 (16.7)	4 (6.1)	
Non-melanoma skin cancer	16 (10.7)	8 (9.5)	8 (12.1)	
Sinonasal	16 (10.7)	11 (13.1)	5 (7.6)	
Thyroid	16 (10.7)	8 (9.5)	8 (12.1)	
Other^d^	17 (11.3)	12 (14.3)	5 (7.6)	
*De novo* presentation, no. (%)	109 (72.7)	63 (75.0)	46 (69.7)	.47
Histology, no. (%)				.02
Squamous cell carcinoma	93 (62.0)	45 (53.4)	48 (72.7)	
Other^e^	57 (38.0)	39 (46.4)	18 (27.3)	
Human papillomavirus associated				.54
No	5 (3.3)	4 (4.8)	1 (1.5)	
Yes	26 (17.3)	14 (16.7)	12 (18.2)	
Not reported	119 (79.3)	66 (78.6)	53 (80.3)	
Pathologic tumor stage, no. (%)				.02
0-2	78 (52.0)	51 (60.7)	27 (40.9)	
3-4	72 (48.0)	33 (39.3)	39 (59.1)	
Pathologic nodal stage, no. (%)				.01
x-1	107 (71.3)	67 (79.8)	40 (60.6)	
2-3	43 (28.7)	17 (20.2)	26 (39.4)	
Positive surgical margins, no. (%)	44 (29.3)	27 (32.1)	17 (25.8)	.39
Extracapsular extension, no. (%)				.09
Absent	75 (50.0)	43 (51.2)	32 (48.5)	
Present	45 (30.0)	20 (23.8)	25 (3)	
Not reported/applicable	30 (20.0)	21 (25.0)	9 (13.6)	
Perineural invasion, no. (%)				.51
Absent	63 (42.0)	36 (42.9)	27 (40.9)	
Present	64 (42.7)	33 (39.3)	31 (47.0)	
Not reported	23 (15.3)	15 (17.9)	8 (12.1)	
Definitive treatment intent, no. (%)^f^	135 (90.0)	83 (98.8)	52 (78.8)	<.001
Total radiation dose, median (IQR), Gy	66.0 (60.0-70.0)	61.2 (60.0-66.0)	69.5 (55.0-70.0)	<.001
Number of radiation fractions, median (IQR)	31.5 (30.0-33.0)	30.0 (30.0-33.0)	33.0 (18.0-33.0)	.047
Re-irradiation setting, no. (%)	14 (9.3)	8 (9.5)	6 (9.1)	.93
Systemic therapy, no. (%)	57 (38.0)	23 (27.4)	34 (51.5)	.002
Time from surgery to FDG-PET/CT, median (IQR), days	35.5 (26.0-51.0)	35.0 (24.5-46.0)	37.0 (28.0-53.0)	.10
Time from FDG-PET/CT to start of radiation, median (IQR), days	17.0 (11.0-27.0)	15.5 (11.0-21.5)	19.5 (9.0-32.0)	.30
Time from surgery to start of radiation, median (IQR), days	54.0 (43.0-69.0)	52.5 (42.0-67.5)	56.5 (44.0-71.0)	.11
Follow-up time, median (IQR), months	21.2 (12.5-46.1)	27.0 (14.4-49.8)	16.0 (8.9-35.7)	.004

Abbreviations: FDG-PET/CT = 18-fluorodeoxyglucose positron emission tomography/computed tomography; IQR = interquartile range.

a Between no plan change and plan change patients.

b Includes Black or African American (*n* = 2), Middle Eastern or North African (*n* = 3), Native Hawaiian or other Pacific Islander (*n* = 1), Multiple Races (*n* = 10), or “Other” (*n* = 25).

c *P *= .02 when comparing oral cavity vs combined all other primary sites.

d Includes unknown primary (*n* = 5), larynx (*n* = 4), mucosal melanoma (*n* = 4), hypopharynx (*n* = 2), and nasopharynx (*n* = 2).

e Includes Papillary thyroid carcinoma (*n *= 12), Carcinoma ex pleomorphic adenoma (*n* = 8), Adenoid cystic carcinoma (*n* = 6), Anaplastic thyroid carcinoma (*n* = 4), Melanoma (*n* = 4), Merkel cell carcinoma (*n* = 3), High-grade/undifferentiated carcinoma (*n* = 2), HPV-related multiphenotypic sinonasal carcinoma (*n *= 2), Lymphoepithelial carcinoma (*n* = 2), Myoepithelial carcinoma (*n* = 2), Odontogenic carcinoma (*n* = 2), Olfactory neuroblastoma (*n* = 2), Salivary ductal carcinoma (*n* = 2), Carcinoma with basaloid features (*n* = 1), Hemangioblastoma (*n* = 1), Malignant peripheral nerve sheath tumor (*n* = 1), Sebaceous cell carcinoma (*n* = 1), Sinonasal undifferentiated carcinoma (*n* = 1), Spindle cell sarcoma (*n* = 1).

f Treatment intent after postoperative FDG-PET/CT.

### Management changes

**Figure 1.**
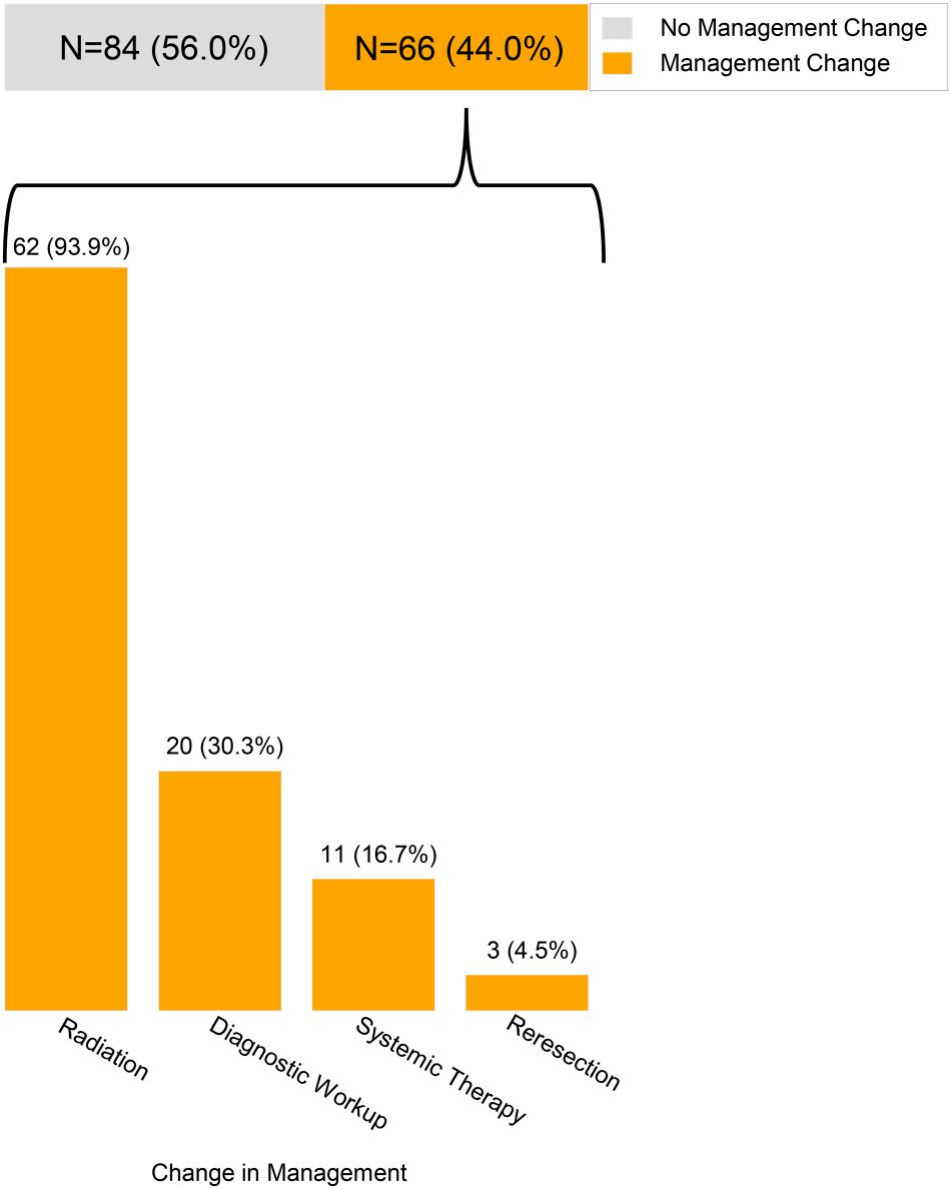
Frequency and types of management changes because of postoperative FDG-PET/CT findings.

A total of 66 (44.0%) patients had any management change because of postoperative FDG-PET/CT findings (Figure 1). Of these, 8 (12.1%) patients had an interval physical exam finding, which prompted the FDG-PET/CT. The majority of these patients had changes to their adjuvant radiotherapy plans (*n* = 62, 93.9% of patients with a management change), including 15 (10.0% of all patients, 22.7% of those with a management change) patients for whom adjuvant radiotherapy was switched to palliative-intent (*n* = 7, 4.7%) or deferred entirely given discovery of significant residual, recurrent, and/or metastatic disease (*n* = 8, 5.3%). Radiotherapy changes included boosting (*n* = 25, 40.3% of patients with a change in radiotherapy), treating a new head and neck area that would not have initially been included in the radiotherapy field (*n* = 11, 17.7%), or both (*n* = 13, 21.0%). Targets of these changes included residual or recurrent disease (*n* = 20, 32.2%), head and neck lymph nodes (*n* = 19, 30.6%), both (*n *= 12, 19.3%), and in 2 (3.3%) patients, definitive radiotherapy to oligometastases. Table 1 shows characteristics of patients who did or did not have a management change because of their postoperative FDG-PET/CT. Patients with a positive postoperative FDG-PET/CT more frequently had SCC histology (72.7% vs 53.4%; *P *= .02), pT3-4 disease (59.1% vs 39.3%; *P *= .02), pN2-3 disease (39.4% vs 20.2%; *P *= .01), and preoperative staging imaging (71.2% vs 47.6%; *P *= .004).

**Figure 2.**
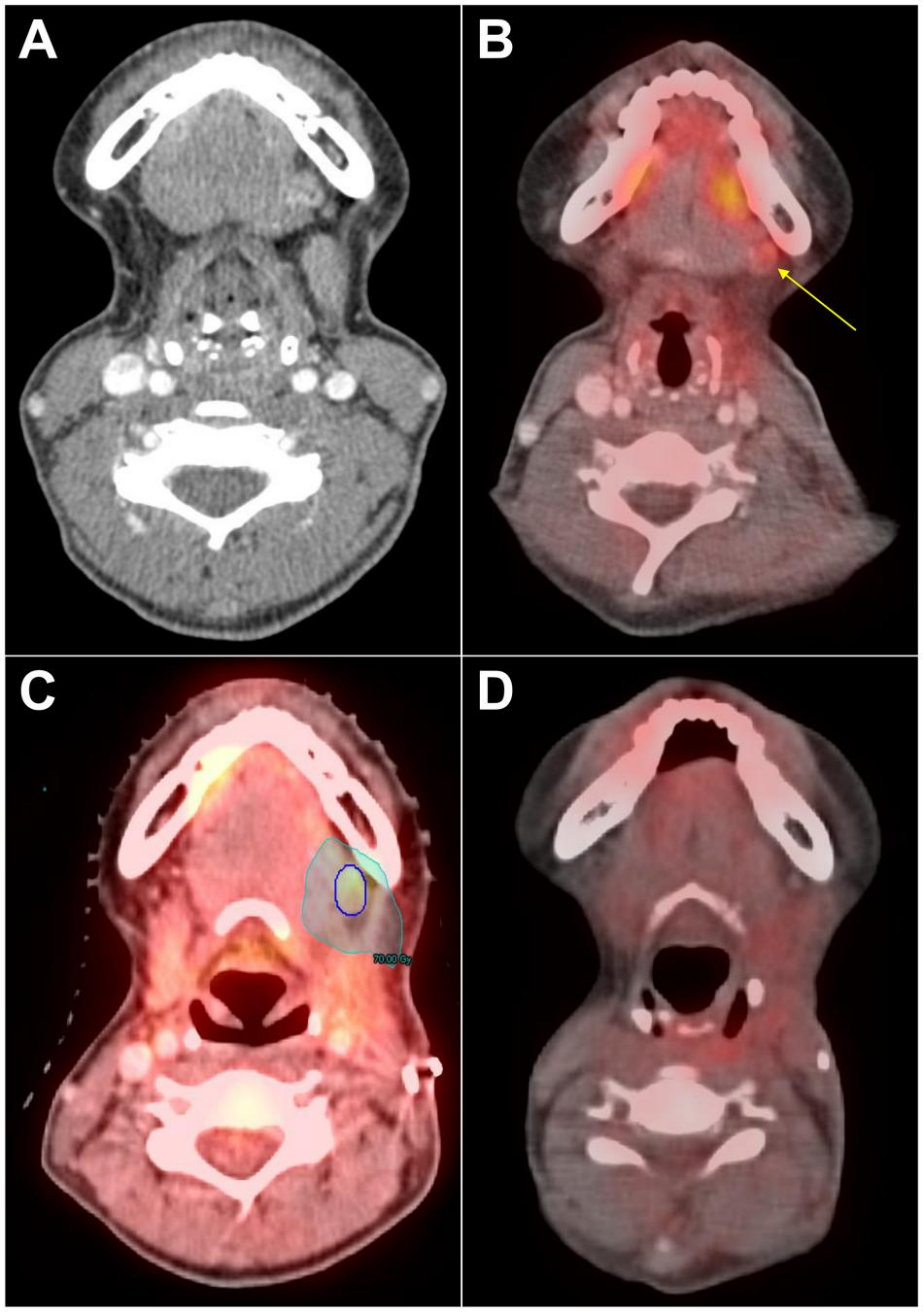
Representative images of a patient who had a management change because of postoperative FDG-PET/CT findings. This patient presented with a left lateral tongue nodule, biopsy of which showed squamous cell carcinoma. Preoperative CT neck and chest with contrast (**A**) showed bilateral, nonspecific neck lymph nodes all <1 cm in short-axis and no gross oral cavity lesion, and neck ultrasound did not find any abnormal lymph nodes. They underwent left partial glossectomy and left neck dissection which showed a 1.5 cm tumor with 0.9 cm depth of invasion, 2/21 lymph nodes positive without extracapsular extension, overall AJCC 8th stage pT2N2b. Additionally, margins were negative, and there was no perineural or lymphovascular invasion. At radiation oncology consult, adjuvant radiotherapy to a maximum dose of 60 Gy without concurrent systemic therapy was recommended, as was FDG-PET/CT, which showed a new, enlarged, FDG-avid left submandibular lymph node (**B**). Subsequent biopsy showed squamous cell carcinoma. Repeat surgery was attempted but the node was unable to be localized intraoperatively, so the patient proceeded with adjuvant radiotherapy to a maximum dose of 70 Gy (**C**) with concurrent cisplatin 100 mg/m^2^ every 3 weeks. Posttreatment FDG-PET/CT showed resolution of the lymph node (**D**).

Eleven (16.7%) patients had systemic therapy added or changed, and 3 (4.6%) patients underwent reresection. Twenty (30.3%) patients underwent additional diagnostic evaluation as a result of the postoperative FDG-PET/CT findings, including 17 (25.8%) who underwent biopsy, of which 14 (82.3%) were positive. In those with abnormal lymph nodes (*n* = 33, 50.0% of all patients with a management change), 24 (72.7%) were ipsilateral, 6 (18.2%) were contralateral, and 3 (9.1%) were bilateral. An example patient case is provided in Figure 2, which highlights how postoperative FDG-PET/CT altered management.

Logistic regression measuring the association between patient factors and the likelihood of having a positive postoperative FDG-PET/CT are presented in Table 2 and Figure S1. Because multiple prior studies evaluated early recurrence in oral cavity cancer specifically^3-5^^,^^8^ and oral cavity was the most represented primary site in this study, we created a binary primary site variable of oral cavity vs all other primary sites combined for the regression analyses. A sensitivity analysis of 1 primary site vs combined all others for other primary sites did not identify any significant associations (Table S1). In the final selected multivariable model, patients with pathologic tumor stage pT3-4 vs pT0-2 (odds ratio [OR] = 3.06, 95% confidence interval [CI] = 1.46 to 6.41; bootstrap CI = 1.37 to 6.53; *P *= .003) or pathologic nodal stage pN2-3 vs pNx-1 (OR = 3.45, 95% CI = 1.52 to 7.84; bootstrap CI = 1.41 to 7.67; *P *= .003) had significantly higher odds of having a positive postoperative FDG-PET/CT.

**Table 2.** Association between clinical factors and likelihood of management change because of postoperative FDG-PET/CT findings.

Variable	Univariable analysis odds ratio (95% confidence interval)	*P-*value	Final selected^a^ multivariable analysis odds ratio (95% confidence interval)	*P*
Increasing age at surgery, years	1.00 (0.98 to 1.02)	.84	1.02 (0.99 to 1.04)	.19
Male sex	1.30 (0.65 to 2.62)	.45	1.13 (0.51 to 2.51)	.77
Self-reported race				
White	Referent	—	Referent	—
Asian	1.08 (0.42 to 2.79)	.87	1.53 (0.52 to 4.46)	.44
Other	1.60 (0.73 to 3.48)	.24	1.77 (0.74 to 4.21)	.20
Not reported	1.35 (0.46 to 3.93)	.58	0.52 (0.09 to 2.99)	.47
Self-reported ethnicity				
Hispanic	Referent	—	Referent	—
Non-Hispanic	0.62 (0.25 to 1.51)	.29	0.45 (0.17 to 1.21)	.12
Not reported	2.14 (0.44 to 10.39)	.35	6.38 (0.73 to 55.81)	.09
Ever tobacco use	1.35 (0.70 to 2.61)	.36	—	—
Increasing year of surgery^b^	1.17 (1.03 to 1.34)	.02	—	—
Had preoperative staging imaging^c^	2.72 (1.37 to 5.39)	.004	—	—
Oral cavity vs all other primary sites	2.37 (1.17 to 4.81)	.02	—	—
*De novo* presentation	0.77 (0.37 to 1.58)	.47	—	—
Squamous cell carcinoma histology	2.31 (1.16 to 4.61)	.02	—	—
Human papillomavirus association^d^	3.43 (0.34 to 35.0)	.30	—	—
Pathologic tumor stage (pT3-4 vs pT0-2)	2.24 (1.16 to 4.31)	.02	3.06 (1.46 to 6.41)^e^	.003
Pathologic nodal stage (pN2-3 vs pNx-1)	2.56 (1.24 to 5.30)	.01	3.45 (1.52 to 7.84)^f^	.003
Positive surgical margins	0.72 (0.36 to 1.50)	.39	—	—
Extracapsular extension				
Absent	Referent	—	Referent	—
Present	1.68 (0.80 to 3.54)	.17	—	—
Not reported/applicable	0.58 (0.23 to 1.42)	.23	—	—
Perineural invasion				
Absent	Referent	—	Referent	—
Present	1.25 (0.62 to 2.52)	.53	—	—
Unknown/not reported	0.71 (0.26 to 1.92)	.51	—	—
Increasing days from surgery to FDG-PET/CT	1.01 (0.99 to 1.03)	.10	—	—

a Covariate selection process is described in the “Methods” section.

b Not included in subsequent variable selection process as this likely reflects changing institutional practice to more frequently obtain postoperative 18-fluorodeoxyglucose positron emission tomography/computed tomography (FDG-PET/CT) over time and is not plausibly related to the likelihood of having a positive postoperative FDG-PET/CT.

c Not included in subsequent variable selection process. The increased odds of a positive postoperative FDG-PET/CT in patients who had preoperative staging imaging is suspected to reflect that patients presenting with high-risk disease more often receive preoperative staging imaging. Thus, the increased odds of a positive postoperative FDG-PET/CT in these patients are therefore likely related to their high-risk disease status and unrelated to having preoperative staging imaging.

d In patients where human papillomavirus association was reported (*n *= 31).

e Bootstrap-generated confidence interval with 500 samples was 1.37 to 6.53.

f Bootstrap-generated confidence interval with 500 samples was 1.41 to 7.67.

Of those with SCC histology, 48 (51.6%) had a management change. Logistic regression of this cohort yielded the same results, whereby pT3-4 vs pT0-2 (OR = 5.74, 95% CI = 2.02 to 16.30; *P *= .001) and pathologic nodal stage pN2-3 vs pNx-1 (OR = 4.14, 95% CI = 1.41 to 12.12; *P *= .01) were the only covariates with significantly higher odds of having a positive postoperative FDG-PET/CT in the final selected multivariate model (Table S2).

### Recurrence

**Figure 3.**
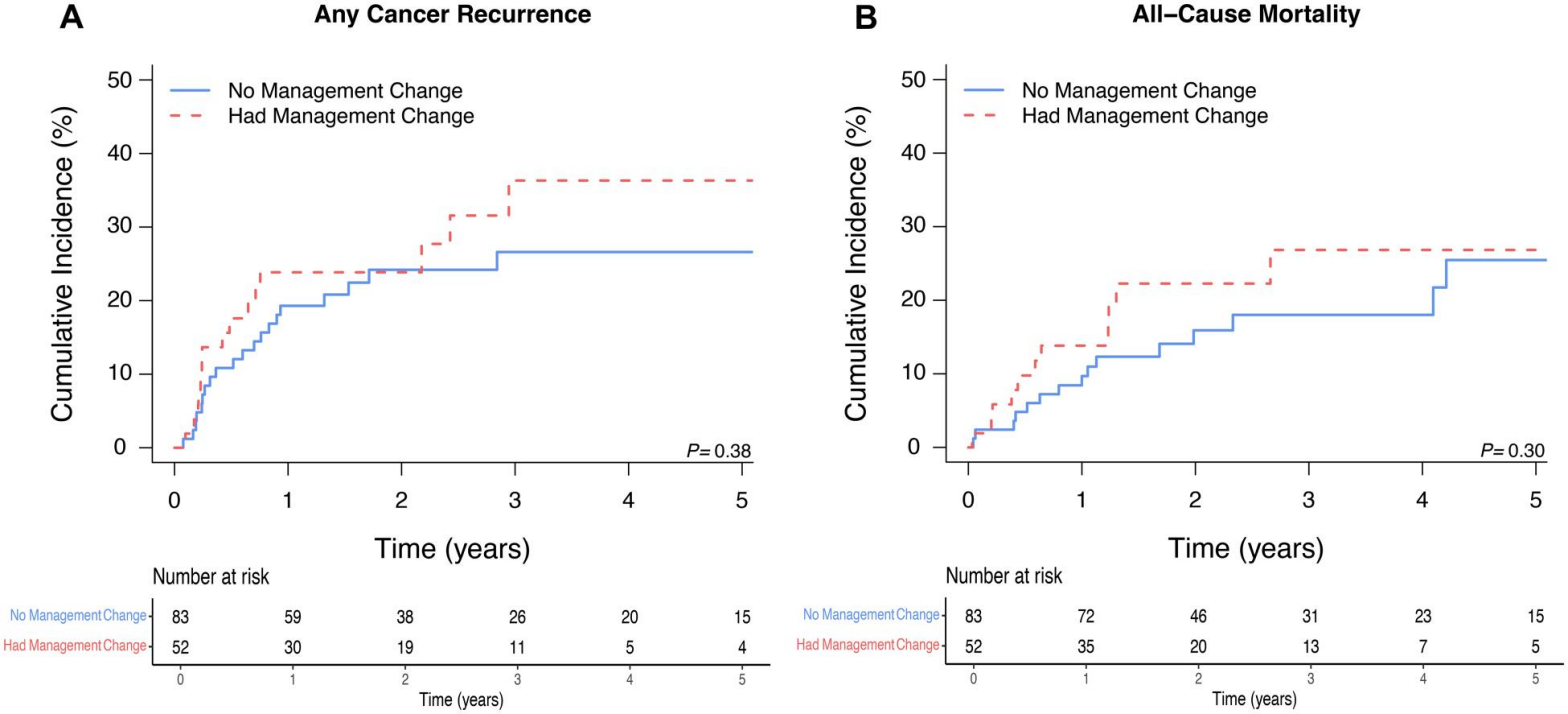
Two-year cumulative incidences of any cancer recurrence (**A**) and all-cause mortality (**B**) in definitively treated patients with a “negative” postoperative FDG-PET/CT by whether postoperative FDG-PET/CT resulted in a management change.

Among patients treated definitively (*n* = 135, 90.0%), 35 (25.9%) experienced any cancer recurrence at a median of 5.9 months (IQR = 2.8-10.8), of which 17 (48.6%) were locoregional only, 12 (34.3%) were distant only, and 6 (17.1%) were both at time of recurrence. The 2-year cumulative incidence of any recurrence was 24.3% (95% CI = 17.1 to 32.1) and was not significantly different between patients with a positive (23.9%; 95% CI = 13.1 to 36.4) vs a negative (24.2%; 95% CI = 15.2 to 34.3) postoperative FDG-PET/CT (*P *= .38) (Figure 3, A). In uni- and selected multivariable competing-risks regression, a positive postoperative FDG-PET/CT was not associated with the risk of any recurrence (univariable subdistribution hazard ratio [SHR] = 1.34, 95% CI = 0.69 to 2.61, *P *= .38; selected multivariable SHR = 0.98, 95% CI = 0.46 to 2.08, *P *= .95).

Among patients with SCC histology and treated definitively (*n* = 80, 53.3%), the 2-year cumulative incidence of any recurrence was 32.1% (95% CI = 21.6 to 43.1) and was not significantly different between patients with a positive (31.4%; 95% CI = 16.8 to 47.2) vs a negative (32.0%; 95% CI = 17.9 to 47.0) postoperative FDG-PET/CT (*P *= .51). In uni- and selected multivariable competing-risks regression, a positive postoperative FDG-PET/CT was not associated with the risk of any recurrence (univariable SHR = 1.29, 95% CI = 0.60 to 2.80, *P *= .43; selected multivariable SHR = 1.08, 95% CI = 0.39 to 2.97, *P *= .89).

### Overall survival

Among patients treated definitively, 29 (19.3%) experienced death from any cause at a median of 12.0 months (IQR = 5.0-23.8). The 2-year cumulative incidence of all-cause mortality was 18.3% (95% CI = 11.8% to 25.9%) and was not significantly different between patients with a positive (22.3%; 95% CI = 11.1 to 35.8) vs a negative (15.9%; 95% CI = 9.6 to 25.3) postoperative FDG-PET/CT (*P *= .30) (Figure 3, B). In uni- and selected multivariable Cox regression, a positive postoperative FDG-PET/CT was not associated with the risk of all-cause mortality (univariable HR = 1.49, 95% CI = 0.72 to 3.06, *P *= .29; selected multivariable HR = 1.42, 95% CI = 0.65 to 3.11, *P *= .38).

Among patients with SCC histology and treated definitively, the 2-year cumulative incidence of all-cause mortality was 25.1% (95% CI = 15.5 to 35.9) and was not significantly different between patients with a positive (32.7%; 95% CI = 16.2 to 50.3) vs a negative (19.5%; 95% CI = 8.9 to 33.2) postoperative FDG-PET/CT (*P *= .27). In uni- and selected multivariable competing-risks regression, a positive postoperative FDG-PET/CT was not associated with the risk of any recurrence (univariable SHR = 1.61, 95% CI = 0.71 to 3.65, *P *= .25; selected multivariable HR = 1.60, 95% CI = 0.73 to 3.49, *P *= .24).

## Discussion

In one of the largest, most diverse studies of the utility of postoperative, pre-adjuvant therapy FDG-PET/CT in patients with head and neck cancer, we found many patients had findings on their postoperative FDG-PET/CT that resulted in a change in management. These changes most frequently occurred in adjuvant radiotherapy plans and included boosting, treating a new site, or both. Beyond radiotherapy, additional diagnostic workup and systemic therapy changes occurred in a considerable proportion of patients, as did transitioning treatment-intent from definitive to palliative, highlighting the clinical utility and versatility of postoperative FDG-PET/CT.

The rate of management changes in the current study is higher than prior studies, which reported rates of 15%-30%,^3-6^ likely for multiple reasons. Our outcome definition differs from prior studies in that we were interested in management change vs residual/recurrent disease. As such, some patients with a “positive” postoperative FDG-PET/CT underwent additional diagnostic workup though ultimately were not found to have residual/recurrent cancer. We also included patients with recurrent cancer as opposed to many of the prior studies. As with prior studies, this was a single-institution study, and our findings may reflect specific institutional practices and patient populations. Nonetheless, this study not only corroborates the previous findings but also further extends these findings in a broader range of head and neck cancer sites as prior studies solely evaluated postoperative FDG-PET/CT in oral cavity cancer.

While promising, our findings should spur multidisciplinary discussions and careful considerations for deciding when, how, and for whom postoperative FDG-PET/CT imaging should be obtained before widespread adoption. Obtaining an FDG-PET/CT can be logistically difficult and may delay starting adjuvant therapy. This challenge may partially explain the median time-to-initiation of adjuvant radiotherapy greater than 6 weeks in this study. Increased time-to-initiation of adjuvant radiotherapy and treatment package time, which are closely related, are both associated with worse outcomes in head and neck cancer,^2^^,^^13-15^ particularly for high-risk patients. Critically, initiation of adjuvant radiotherapy or treatment package time should not be markedly prolonged to coordinate a postoperative FDG-PET/CT, especially in patients for whom adjuvant treatment needs to start urgently.

Admittedly, obtaining a postoperative FDG-PET/CT increases upfront health-care costs for the additional scan from which more than half of patients will not see an immediate benefit. However, these scans hold potential to markedly reduce long-term costs for cancer centers by guiding more appropriate management decisions. For example, when postoperative FDG-PET/CT identifies new metastatic disease, a more appropriate shorter, palliative-intent treatment would have a substantial cost reduction compared to longer, more resource-intense definitive treatment.^6^^,^^16^ In the initial diagnosis and post-(chemo)radiotherapy surveillance settings, FDG-PET/CT is cost-effective primarily because it results in fewer, often unbeneficial interventions.^17-20^ Similarly, FDG-PET/CT in the postoperative, pre-adjuvant therapy setting may be a valuable practice through reductions in unnecessarily aggressive treatments, as well as possibly through improvements in clinical outcomes from updated treatment plans.

It may be reasonable to presume that a postoperative FDG-PET/CT provides a clinical benefit, though the current study does not include a comparator group of patients who did not undergo a postoperative FDG-PET/CT to confirm or quantify this. One prior study demonstrated a disease-free and overall survival benefit associated with postoperative FDG-PET/CT in patients with oral cavity SCC with intermediate risk features,^4^ though this association was not seen in a different study.^5^ Even so, there are benefits of postoperative FDG-PET/CT that can be realized even in the absence of such a comparison, such as improved prognostication and relatedly, changing to palliative-intent treatment when indicated, which occurred in 10% of all patients (22.7% of those with a management change) in this study. Prospective, randomized studies may prove challenging in this clinical setting, but large, retrospective, multi-institutional, pooled data may help further define the utility and efficacy of obtaining postoperative FDG-PET/CTs in patients with head and neck cancer.

Historically, concern for postoperative inflammatory changes^9^ also limited extensive use of postoperative FDG-PET/CT. In this study, postoperative FDG-PET/CT had a positive predictive value (PPV) 82.3%, although only a minority of patients underwent biopsy of suspicious findings due to patient declination, inaccessibility, or urgency to start adjuvant treatment. Prior studies of postoperative FDG-PET/CT reported PPVs of 40%-60%.^3^^,^^4^^,^^6^ FDG-PET/CTs in the post-(chemo)radiotherapy setting demonstrate comparable PPVs for detecting residual primary site or neck disease,^20-22^ albeit is obtained 3 months after completion of treatment. Although there is a real risk of unnecessary procedures, delays, and overtreatment with postoperative FDG-PET/CT, these data highlight that the false-positive rate may be lower and not as limiting to optimizing treatment as previously thought. Still, the low biopsy rate in the current trial limits the ability to determine whether the postoperative FDG-PET/CT findings were true vs false positives. Conversely, the limits of FDG-PET/CT sensitivity should be noted, and even a negative postoperative FDG-PET/CT cannot absolutely rule out residual/recurrent disease. Ultimately, careful and ideally multidisciplinary review along shared decision-making may allow for appropriate triaging of patients toward biopsy or other diagnostic evaluations vs treatment based on postoperative FDG-PET/CT findings alone. Furthermore, given the expertise required to safely boost or incorporate new targets in the radiotherapy plan, treating abnormal findings on postoperative FDG-PET/CT should be performed cautiously in less-experienced or resourced settings.

This study has multiple limitations. It is a retrospective study without standardized protocols for obtaining a postoperative FDG-PET/CT, treatment or follow-up. Only about 60% of patients had preoperative staging imaging, and thus, postoperative FDG-PET/CT may have identified disease that was unrecognized prior to surgery. However, for many head and neck cancers, preoperative staging imaging, including FDG-PET/CT, is recommended to be obtained only as clinically indicated.^1^ These patients’ initial disease may have been deemed low-risk enough to not warrant preoperative staging imaging, or perhaps other factors, such as the desire to proceed with surgery quickly, resulted in omitting preoperative staging imaging. For comparison, the aforementioned studies of early recurrence reported preoperative imaging rates of 60%-100%. Additionally, we found that having preoperative staging imaging was associated with increased risk of having a positive postoperative FDG-PET/CT, likely reflective of confounding by indication.^23^ Selection bias may have additionally contributed to the results as we excluded patients for whom postoperative FDG-PET/CT was desired but unable to be obtained because of access to care limitations. The study population is relatively small, heterogeneous, and included certain cancers which may have different treatment paradigms from more general head and neck cancers, such as thyroid cancer, which may limit the external validity of the study.

In patients with head and neck cancer who underwent surgery, postoperative, pre-adjuvant therapy FDG-PET/CT can be abnormal and may help guide adjuvant management. When practical to obtain without introducing treatment delays, postoperative FDG-PET/CT may have clinical utility though requires careful interpretation due to the risks of false positives resulting in potentially unnecessary procedures or overtreatment.

## Supplementary Material

pkaf077_Supplementary_Data

## Data Availability

The data underlying this article are available in the article and in its online supplementary material. Individual patient data are stored in an institutional electronic medical record system and will be shared upon request in de-identified, HIPAA-complaint form.
