# Supplementary material for: Impact of postoperative fluorodeoxyglucose positron emission tomography/computed tomography on adjuvant head and neck cancer treatment
Source: JNCI Cancer Spectr. 2025 Jul 23;9(4):pkaf077. doi: 10.1093/jncics/pkaf077 (PMC12349773; doi:10.1093/jncics/pkaf077)
Supplement: pkaf077_Supplementary_Data [file pkaf077_supplementary_data.zip › HNC Postop PET_Supplementary Material Revised.pdf]

Supplementary Material

Supplementary Methods

In the logistic regression analysis for identifying patient factors associated with having a positive postoperative FDG-PET/CT, variables with a $p<0.2$ in univariable analysis and deemed plausibly associated with the likelihood of having a positive postoperative FDG-PET/CT were first added into a multivariable logistic regression. Then, variables with a $p>0.05$ were removed one-by-one in order of decreasing *p*-value until only variables with $p<0.05$ remained. Finally, age, sex, race, and ethnicity were added into the multivariable logistic regression model to evaluate the impact of sociodemographic factors on the association between selected patient factors and having a positive postoperative FDG-PET/CT.

In the univariable logistic regression analysis, increasing year of surgery was significantly associated with an increased risk of having a postoperative FDG-PET/CT. This variable was not included in subsequent variable selection process as this likely reflects changing institutional practice to more frequently obtain postoperative FDG-PET/CT over time, and is not plausibly related to the likelihood of having a positive postoperative FDG-PET/CT. Additionally, having preoperative staging imaging was significantly associated with an increased risk of having a positive postoperative FDG-PET/CT on univariable logistic regression. This factor was not included in subsequent variable selection process as the increased odds of a positive postoperative FDG-PET/CT in patients who had preoperative staging imaging is suspected to reflect that patients presenting with higher-risk disease more often receive preoperative staging imaging. Thus, the increased odds of a positive postoperative FDG-PET/CT in these patients are therefore likely related to their higher-risk disease status and unrelated to having preoperative staging imaging.

In the Fine-Gray competing-risks and Cox proportional hazards regression analyses for cancer recurrence and overall survival, respectively, the covariate selection process was the same as for the logistic regression analysis described above, though incorporated additional covariates including receipt of systemic therapy, receipt of reirradiation, radiotherapy dose, and time-to-initiation of adjuvant radiotherapy from surgery as a continuous variable. Additionally, in the analysis of all patients, the postoperative FDG-PET/CT covariate (positive or negative) was incorporated into the final selected multivariable regression model regardless of its *p*-value on univariable or multivariable analysis. As in the logistic regression analysis, year of surgery and having preoperative staging imaging were not included in the Fine-Gray or Cox regression covariate selection process.

Table S1: Association between individual primary head and neck cancer site and likelihood of Management Change because of postoperative FDG-PET/CT findings.

Primary Site	Univariable Analysis Odds Ratio (95% Confidence Interval)	*P*-value
Oral Cavity versus All Other Primary Sites	2.37 (1.17-4.81)	0.02
Oropharyngeal versus All Other Primary Sites	0.95 (0.37-2.40)	0.91
Salivary Gland versus All Other Primary Sites	0.32 (0.10-1.03)	0.057
Non-Melanoma Skin Cancer versus All Other Primary Sites	1.31 (0.46-3.70)	0.61
Sinonasal versus All Other Primary Sites	0.54 (0.18-1.65)	0.28
Thyroid versus All Other Primary Sites	1.31 (0.46-3.70)	0.61
Other[1] versus All Other Primary Sites	0.49 (0.16-1.47)	0.21

[1]Includes unknown primary (n=5), larynx (n=4), mucosal melanoma (n=4), hypopharynx (n=2), and nasopharynx (n=2).

Table S2: Sensitivity analysis of association between clinical factors and likelihood of management change because of postoperative FDG-PET/CT findings in patients with squamous cell carcinoma histology (n=93).

Variable	Univariable Analysis Odds Ratio (95% Confidence Interval)	*P*-value	Final Selected[1] Multivariable Analysis Odds Ratio (95% Confidence Interval)	*P*-value
Increasing Age at Surgery, years	0.99 (0.97-1.02)	0.65	1.01 (0.97-1.04)	0.73
Male Sex	0.97 (0.38-2.49)	0.95	0.73 (0.23-2.32)	0.60
Self-Reported Race				
White	Referent	--	Referent	--
Asian	0.82 (0.24-2.78)	0.75	0.89 (0.19-4.19)	0.88
Other	2.35 (0.87-6.36)	0.09	3.08 (0.94-10.04)	0.06
Not Reported	1.48 (0.39-5.71)	0.57	***	0.97
Self-Reported Ethnicity				
Hispanic	Referent	--	Referent	--
Non-Hispanic	0.95 (0.28-3.21)	0.93	0.74 (0.18-3.02)	0.68
Not Reported	3.50 (0.51-24.27)	0.20	***	0.97
Ever Tobacco Use	1.05 (0.46-2.36)	0.91	--	--
Increasing Year of Surgery[2]	1.13 (0.96-1.33)	0.13	--	--
Had Preoperative Staging Imaging[3]	6.13 (2.17-17.27)	0.0006	--	--
Oral Cavity versus All Other Primary Sites	1.95 (0.85-4.45)	0.11	--	--
De Novo Presentation	0.99 (0.40-2.42)	0.98	--	--
Human Papillomavirus Association[4]	2.75 (0.25-30.51)	0.41	--	--
Pathologic Tumor Stage (pT3-4 versus pT0-2)	3.38 (1.43-7.96)	0.005	5.74 (2.02-16.30)	0.001

Pathologic Nodal Stage (pN2-3 versus pNx-1)	2.75 (1.15-6.56)	0.02	4.14 (1.41-12.12)	0.01
Positive Surgical Margins	1.33 (0.50-3.55)	0.57	--	--
Extracapsular Extension				
Absent	Referent	--	--	--
Present	1.75 (0.72-4.25)	0.22	--	--
Not Reported/ Applicable	0.54 (0.12-2.41)	0.42	--	--
Perineural Invasion				
Absent	Referent	--	--	--
Present	1.51 (0.65-3.49)	0.54	--	--
Unknown/Not Reported	***	0.97	--	--
Increasing Days from Surgery to FDG-PET/CT	1.01 (0.99-1.03)	0.27	--	--

[1]Covariate selection process is described in the Methods section.

[2]Not included in subsequent variable selection process as this likely reflects changing institutional practice to more frequently obtain postoperative FDG-PET/CT over time, and is not plausibly related to the likelihood of having a positive postoperative FDG-PET/CT.

[3]Not included in subsequent variable selection process. The increased odds of a positive postoperative FDG-PET/CT in patients who had preoperative staging imaging is suspected to reflect that patients presenting with higher-risk disease more often receive preoperative staging imaging. Thus, the increased odds of a positive postoperative FDG-PET/CT in these patients are therefore likely related to their higher-risk disease status and unrelated to having preoperative staging imaging.

[4]In patients where human papillomavirus association was reported (n=31).

***Unable to calculate due to insufficient event numbers (i.e., all patients in subgroup had 0 or all management changes).

Figure S1: Forest Plot of association between clinical factors and likelihood of management change because of postoperative FDG-PET/CT findings.

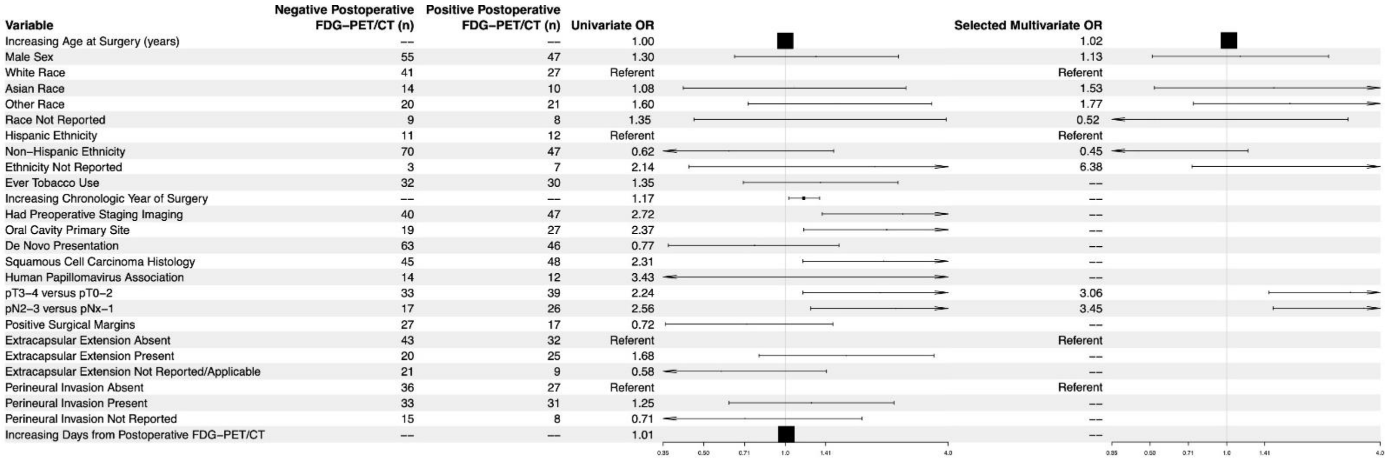

Variable	Negative Postoperative FDG–PET/CT (n)	Positive Postoperative FDG–PET/CT (n)	Univariate OR	Selected Multivariate OR
Increasing Age at Surgery (years)	---	---	1.00	1.02
Male Sex	55	47	1.30	1.13
White Race	41	27	Referent	Referent
Asian Race	14	10	1.08	1.53
Other Race	20	21	1.60	1.77
Race Not Reported	9	8	1.35	0.52
Hispanic Ethnicity	11	12	Referent	Referent
Non–Hispanic Ethnicity	70	47	0.62	0.45
Ethnicity Not Reported	3	7	2.14	6.38
Ever Tobacco Use	32	30	1.35	---
Increasing Chronologic Year of Surgery	---	---	1.17	---
Had Preoperative Staging Imaging	40	47	2.72	---
Oral Cavity Primary Site	19	27	2.37	---
De Novo Presentation	63	46	0.77	---
Squamous Cell Carcinoma Histology	45	48	2.31	---
Human Papillomavirus Association	14	12	3.43	---
pT3–4 versus pT0–2	33	39	2.24	3.06
pN2–3 versus pNx–1	17	26	2.56	3.45
Positive Surgical Margins	27	17	0.72	---
Extracapsular Extension Absent	43	32	Referent	Referent
Extracapsular Extension Present	20	25	1.68	---
Extracapsular Extension Not Reported/Applicable	21	9	0.58	---
Perineural Invasion Absent	36	27	Referent	Referent
Perineural Invasion Present	33	31	1.25	---
Perineural Invasion Not Reported	15	8	0.71	---
Increasing Days from Postoperative FDG–PET/CT	---	---	1.01	---
